# Reconstruction of the Steroid 1(2)-Dehydrogenation System from *Nocardioides simplex* VKM Ac-2033D in *Mycolicibacterium* Hosts

**DOI:** 10.3390/microorganisms11112720

**Published:** 2023-11-07

**Authors:** Svetlana R. Fufaeva, Dmitry V. Dovbnya, Tanya V. Ivashina, Andrei A. Shutov, Marina V. Donova

**Affiliations:** G. K. Skryabin Institute of Biochemistry and Physiology of Microorganisms, “Pushchino Scientific Center for Biological Research of the Russian Academy of Sciences”, 142290 Pushchino, Russia; sfufaeva@list.ru (S.R.F.); anagoge@rambler.ru (D.V.D.); ivashina@ibpm.ru (T.V.I.); w___w@rambler.ru (A.A.S.)

**Keywords:** biotransformation, *Mycolicibacterium*, recombinant strains, steroid, 3-ketosteroid-1(2)-dehydrogenase, hydrocortisone, prednisolone

## Abstract

Microbial 1(2)-dehydrogenation of 3-ketosteroids is an important basis for the production of many steroid pharmaceuticals and synthons. When using the wild-type strains for whole cell catalysis, the undesirable reduction of the 20-carbonyl group, or 1(2)-hydrogenation, was observed. In this work, the recombinant strains of *Mycolicibacterium neoaurum* and *Mycolicibacterium smegmatis* were constructed with blocked endogenous activity of 3-ketosteroid-9α-hydroxylase, 3-ketosteroid-1(2)-dehydrogenase (3-KSD), and expressing 3-KSD encoded by the gene *KR76_27125* (*kstD2*_NS_) from *Nocardioides simplex* VKM Ac-2033D. The in vivo activity of the obtained recombinant strains against phytosterol, 6α-methyl-hydrocortisone, and hydrocortisone was studied. When using *M. smegmatis* as the host strain, the 1(2)-dehydrogenation activity of the constructed recombinant cells towards hydrocortisone was noticeably higher compared to those on the platform of *M. neoaurum*. A comparison of the strengths of inducible acetamidase and constitutive *hsp60* promoters in *M. smegmatis* provided comparable results. Hydrocortisone biotransformation by *M. smegmatis* BD/pMhsp_*k* expressing *kstD2_NS_* resulted in 95.4% prednisolone yield, and the selectivity preferred that for *N. simplex.* Mycolicibacteria showed increased hydrocortisone degradation at 35 °C compared to 30 °C. The presence of endogenous steroid catabolism in *Mycolicibacterium* hosts does not seem to confer an advantage for the functioning of KstD2_NS_. The results allow for the evaluation of the prospects for the development of simple technological methods for the selective 1(2)-dehydrogenation of 3-ketosteroids by growing bacterial cells.

## 1. Introduction

Microbial 1,2-dehydrogenation is one of the most important reactions of structural modification of steroids [1] that plays a key role in the preparation of therapeutic steroids such as prednisone, prednisolone [2], 6α-metyl-prednisolone, as well as important synthons including androstadienedione (ADD), 20-hydroxymethylpregna-1,4-diene-3-one (HMPD) [3], 21-acetoxypregna-1(2),4(5),9(11),16(17)-tetraene-21-ol-3,20-dione (“tetraene”) [4], that are used in the syntheses of corticoids [5], anabolics [1] and anticancer drugs [6]. The presence of a C1-C2-double bond in ring A of the steroid core enhances the anti-inflammatory effect of the therapeutics and reduces side effects in comparison with their 1(2)-saturated analogues [7].

The ability to carry out 1(2)-dehydrogenation was demonstrated for the representatives of various microbial phyla; however, the highest level of activity was shown for actinobacteria of the genera *Nocardioides*, *Mycolicibacterium*, *Nocardia*, and *Rhodococcus* [1,8]. Among them, a special place is occupied by industrial strains of soil actinobacteria *Nocardioides simplex* (previously classified as *Mycobacterium globiforme* 193, *Arthrobacter globiformis*, *Corynebacterium simplex*, *Arthrobacter simplex*, and *Pimelobacter simplex*), since their resting, processed, or immobilized cells are able to effectively dehydrogenate natural and synthetic steroid substrates [8,9].

In actinobacteria, 3-ketosteroid-1(2)-dehydrogenase (3-KSD, EC 1.3.99.4) is mainly an intracellular enzyme [10] and can be associated with the membrane (*N. simplex*) [11,12] or localized in the cytosol (*Rhodococcus erythropolis*) [13]. The enzyme contains a flavin prosthetic group (FAD), which is reduced upon dehydrogenation of the steroid substrate and transfers electrons to the respiratory chain [10,11,14].

**Figure 1:**
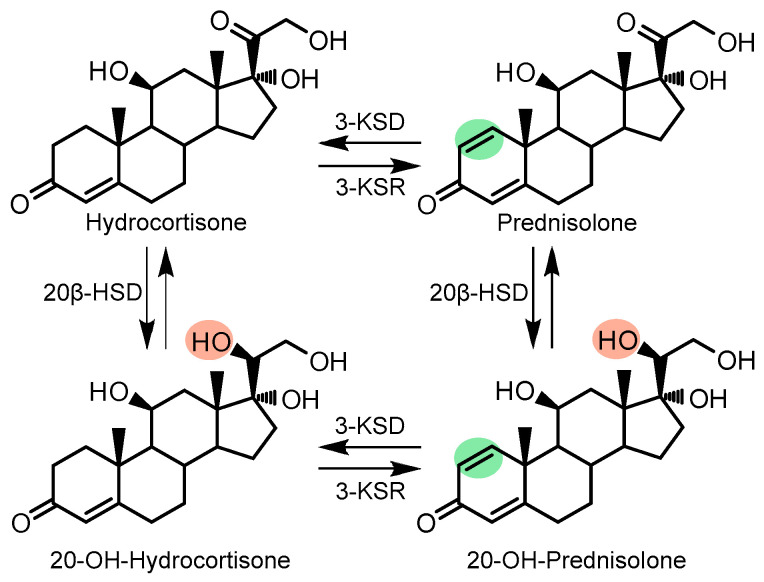
Target reactions of 1(2)-dehydrogenation (green) and undesirable 1(2)-hydrogenation and 20β-reduction (orange) of hydrocortisone by *N. simplex* Ac-2033D [18]. Enzymes: 3-KSD—3-ketosteroid-1(2)-dehydrogenase, 3-KSR—3-ketosteroid-1(2)-reductase, 20β-HSD—20β-hydroxysteroid-dehydrogenase.

In the genome of *N. simplex* VKM Ac-2033D (GenBank: CP009896.1), five genes were identified that putatively encode 3-KSDs [15]. The genes are distributed throughout the bacterial chromosome, expressed under the control of various transcriptional regulators, and differ significantly in the level of expression in response to steroids and sterols [16]. Induction of 3-KSD by androstenedione (AD) (*Nocardia rhodochrous*) [17], cortisone (*Corynebacterium simplex*) [18], and cortisone-21-acetate (AcC) (*N. simplex*) [9] has been shown. However, when using the whole cells, the undesirable side activities observed complicate further purification of the final crystalline 1(2)-dehydrogenated steroids. As shown for *N. simplex* and related actinobacteria, the most significant undesirable activity leads to the reduction of the 20-carbonyl group of steroids to the 20β-hydroxy group [8,9,12,19] in both the 1(2)-saturated steroid substrate and the 1(2)-dehydrogenated product (Figure 1). This modification is presumably associated with the activity of NAD(P)H-dependent 3α(or 20β)-hydroxysteroid dehydrogenases (20β-HSD) previously found in strains of *Streptomyces hydrogenans* [20] and *Bacillus megaterium* [21], whose gene orthologues are also present in the genome of *N. simplex* Ac-2033D [15].

The problem can be solved either by suppressing the undesirable 20β-reducing activity in *N. simplex* or by heterologous expression of the 3-KSD genes in strains that do not have such activity. Noteworthy, the engineering of *N. simplex* strains is complicated by the current lack of appropriate genetic tools [22,23]. On the contrary, the expression of 3-KSD genes in strains that do not have endogenous steroid catabolism (*E. coli* [5,23,24,25,26,27,28,29,30], *Bacillus subtilis* [5,25,31], *Corynebacterium crenatum* [32], and *Pichia pastoris* [33]) made it possible to effectively produce target steroids, but mainly in the presence of exogenous electron acceptors (EEA). For example, the expression of the synthetic PrKstD gene from *Propionibacterium* sp. in *E. coli* BL21(DE3) effectively converted 40–70 g/L of hydrocortisone to prednisolone with a 92.5–95.5% yield [30].

There are also reports indicating the increased production of 1(2)-dehydro-steroids by steroid-transforming actinobacteria bearing additional alleles of 3-KSDs genes [34,35]. For instance, expression in *M. neoaurum* NwIB-01 of either an additional copy of its own 3-KSD gene or heterologous expression of the 3-KSD gene from *A. simplex* 156 (IFO12069) led to an increase in the yield of ADD during phytosterol biotransformation [34]. The introduction of two additional copies of the genes of its own 3-KSD into the loci of the 16S rRNA genes of the strain *Arthrobacter simplex* 156 provided a 1.5-fold increase in the rate of conversion of cortisone acetate as compared with the initial strain [35].

In this work, recombinant strains of *M. neoaurum* and *M. smegmatis* were constructed that lack endogenous 3-ketosteroid-1(2)-dehydrogenase activity and express the *kstD2* gene coding for 3-ketosteroid-1(2)-dehydrogenase from *Nocardioides simplex* VKM Ac-2033D (KstD2_NS_; GenBank: AIY19529.1) under the control of two different promoters; the in vivo activity of heterologous KstD2_NS_ against model steroid substrates and undesirable activities of recombinant cells were studied in comparison with *N*. *simplex*.

## 2. Materials and Methods

### 2.1. Materials

The following media components were used: yeast extract, soy peptone (Himedia, India); bacto-tryptone, corn steep solids, acetamide, menadione, randomly methylated β-cyclodextrin Cavasol W7 M1.8 (MCD) (Merck, Darmstadt, Germany); bacto agar (Panreac, Barcelona, Spain); other components were purchased from local manufacturers (RF). Cortisol (hydrocortisone) (98% purity) was purchased from Sanofi (Paris, France), kanamycin sulfate and hygromycin B were purchased from CDH (India); and 6α-methyl-hydrocortisone was provided by Symbiotec (Indore, India). Restriction endonucleases, T4 phage DNA ligase, and agarose were purchased from Thermo Fisher Scientific (Waltham, MA, USA), Taq polymerase from Alpha Ferment (Moscow, Russia), Q5 DNA polymerase from New England BioLabs (Ipswich, MA, USA), and lysozyme from Merck (Germany). Organic solvents for HPLC were purchased from Panreac (Barcelona, Spain). Analytical standards for steroid compounds were purchased from Steraloids (Newport, RI, USA) and Merck (Darmstadt, Germany).

### 2.2. Microorganisms and Cultivation

The bacterial strains used are listed in Table 1.

Actinobacteria were cultured in MYCB nutrient medium [36] supplemented with 1 g/L Tween 80 at 30 °C (*N. simplex*, *M. neoaurum*) or supplemented with 3 g/L Tween 80 at 37 °C (*M. smegmatis*). *E. coli* bacteria were grown on LB medium [37]. Culture growth was assessed gravimetrically by dry cell weight (DCW) collected from 10 mL of culture broth. Recombinant bacterial strains were cultivated in the presence of kanamycin sulfate (Km)—20 μg/mL or hygromycin B (Hyg)—100 μg/mL.

**Table 1:** The strains and plasmids used in this study.

Strains and Plasmids	Description	Source
*E. coli* DH5α	Strain for maintenance and amplification of plasmids	Thermo Fisher Scientific (USA)
*Nocardioides simplex* VKM Ac-2033D	Strain producing 1,2-dehydrosteroids, source of *kstD2*_NS_ (*KR76_27125*)	All-Russian Collection of Microorganisms (VKM)
*Mycolicibacterium neoaurum * NRRL B-3805∆*kstD*	Sterol-transforming strain producing AD, *kstD* knockouted	Dr. M. Smith, University of York, York, UK [38].
*Mycolicibacterium smegmatis* BD	*M. smegmatis* mc^2^ 155, *kshB* and *kstD* knockouted	Karpov et al., 2022 [39]
*Mycolicibacterium neoaurum * NRRL B-3805∆*kstD*/pMVT61	*M. neoaurum* NRRL B-3805∆*kstD* with pMVT61 plasmid	This study
*Mycolicibacterium neoaurum * NRRL B-3805∆*kstD*/pMami_*k*	*M. neoaurum* NRRL B-3805∆*kstD* with pMami_*k* plasmid	This study
*Mycolicibacterium smegmatis* BD/pMVT61	*M*. *smegmatis* BD with pMVT61 plasmid	This study
*Mycolicibacterium smegmatis* BD/pMami_*k*	*M*. *smegmatis* BD with pMami_*k* plasmid	This study
*Mycolicibacterium smegmatis* BD/pMV261-N	*M*. *smegmatis* BD with pMV261-N plasmid	This study
*Mycolicibacterium smegmatis* BD/pMhsp_*k*	*M*. *smegmatis* BD with pMhsp_*k* plasmid	This study
pSMT3-M	*E. coli*—*Mycobacterium* high-copy shuttle vector, Hyg^R^, P*_hsp60_*, 5.7 kb	Carroll et al., 2010 [40]
pSMT3-MN	pSMT3-M with *Nde*I site introduced to the polylinker	This study
pMVT61	*E. coli*—*Mycobacterium* low-copy shuttle vector, Km^R^, inducible acetamidase promoter (P*_ami_*), C-terminal His-tag, 8.0 kb	Karpov et al., 2022 [39]
pMV261	*E. coli*—*Mycobacterium* low-copy shuttle vector, Km^R^, P*_hsp60_*, 4.4 kb	Stover et al., 1991 [41]
pMV261-N	pMV261 with *Nde*I site introduced to the polylinker	This study
pSM_*k*	pSMT3-MN with *kstD2*_NS_ gene	This study
pMami_*k*	pMVT61 with *kstD2*_NS_ gene	This study
pMhsp_*k*	pMV261-N with *kstD2*_NS_ gene	This study

### 2.3. Construction of Recombinant Plasmids

Shuttle (*E. coli*—*Mycolicibacterium*) plasmids pMVT61, pSMT3-MN, and pMV261-N were used as expression vectors (Table 1). To obtain the pMV261-N vector, a DNA fragment (420 bp) from the pSMT3-MN plasmid containing the *hsp60* promoter and the site of the *NdeI* restriction endonuclease was cloned into the pMV261 plasmid between the *Xba*I and *Hin*dIII sites (Figure S1).

To express *kstD2*_NS_ in *Mycolicibacterium* cells, recombinant plasmids pMhsp_*k*, pMami_*k*, and pSM_*k* were constructed. For cloning in the pSMT3-MN vector, the gene *kstD2*_NS_ was amplified from the total DNA of *N. simplex* VKM Ac-2033D with the kstD2nsf/kstD2nsr primer pair (Table S1). In the case of the vector pMVT61, a three-primer PCR method was used with primers kstD2nf2/kstD2nf3/kstD2nr (Table S1). At the same time, to prevent the fusion of the gene ORF with the 6xHis coding sequence and the TEV-protease recognition site, the TAA termination codon preceding the ATG translation start codon at a distance of six nucleotides upstream was introduced into the kstD2nf2 primer. The *kstD2*_NS_ gene was cloned at the *Nde*I-*Hin*dIII sites to obtain pMami_*k* and pSM_*k* (Figure S1). The pMhsp_*k* plasmid was obtained by subcloning *kstD2*_NS_ from the pMami_*k* plasmid into the vector pMV261-N at the *Nde*I-*Hin*dIII sites (Figure S1). The presence of the target insert in the plasmids was confirmed by PCR.

The nucleotide sequence of the *kstD2* gene was validated by sequencing using primers kstD2_1, kstD2_2, kstD2_3, T1R_r, and Phsp60_f. The nucleotide sequence of the *hsp60* promoter in the vector pMV261-N was sequenced using the primers Phsp60_f and T1R_r (Table S1).

### 2.4. Total DNA Isolation

The cells of *N. simplex* VKM Ac-2033D at the early stationary growth phase (2 mL) were harvested by centrifugation and re-suspended in 360 µL of the solution composed of 25 mM Tris-HCl (pH 8.0) and 10 mM EDTA. Then, lysozyme was added to a final concentration of 1 mg/mL, and the mixture was incubated for 30 min at room temperature. Then, 10% (*w*/*v*) SDS to a final concentration of 1% (*w*/*v*) and proteinase K solution to a final concentration of 400 μg/mL were added. The mixture was incubated at 56 °C for 30 min, then supplemented with RNAse A to a final concentration of 100 μg/mL and incubated for 30 min more. DNA was extracted sequentially with equal volumes of phenol saturated with 100 mM Tris-HCl (pH 8.0), with a mixture of phenol, chloroform, and isoamyl alcohol (25:24:1), supplemented with 0.1 volume of 3.0 M potassium acetate (pH 5.3), and precipitated with 0.7 volumes of isopropyl alcohol. The DNA pellet was washed twice with 750 µL of 70%, once with 96% ethanol, dissolved in TE buffer, heated at 56 °C for 1 h, and stored at −20 °C. The concentration and purity of DNA were determined, respectively, at 260 nm and by absorbance ratio at 260/280 nm using a NanoPhotometer TM P-Class spectrophotometer (Implen, München, Germany).

### 2.5. Isolation of Plasmid DNA

Plasmid DNA from *E. coli* was isolated using the QIAprep Spin Miniprep Kit and the QIAGEN Plasmid Mini Kit (QIAGEN, Germantown, MD, USA) according to the manufacturer’s recommendations. Plasmid DNA from mycolicibacteria was isolated by alkaline lysis with modifications. Cells were grown on MYC-ET medium (the same composition as MYCB, but supplemented with 15 g/L Tween 80 and 15 g/L glycine). The cells were sequentially treated with lysozyme (100 μg/mL, 40 min), then 1% SDS solution with 200 mM NaOH (5 min), and neutralized with 3 M potassium acetate (pH 5.5). Plasmid DNA was precipitated with isopropanol and then washed with 70% ethanol.

### 2.6. Electrotransformation of Mycolicibacterium Cells

To obtain electrocompetent cells, mycolicibacteria were grown in 5 mL of MYCB medium at 200 rpm at 30 °C for 48 h (*M. neoaurum* NRRL B-3805∆*kstD*) or at 37 °C for 24 h (*M. smegmatis* BD). Then, 2 mL of the resulting culture was added to 50 mL of MYC-ET medium and grown to OD_600_ 0.6–0.8. The culture was cooled in an ice bath for 30 min. Cells were harvested by centrifugation at 4000× *g* (15 min, 4 °C). The precipitate was washed successively with 25 mL of chilled deionized water (15 min, 4000× *g*, 4 °C) and 10% glycerol (20 min, 4000× *g*, 4 °C). Then, cells were suspended in 1 mL of a 10% glycerol solution in deionized water, aliquoted, and stored at −70 °C. Electroporation of electrocompetent cells was carried out according to [38].

### 2.7. SDS-PAGE Analysis of Proteins

Recombinant 36 h *Mycolicibacterium* cells after induction with acetamide during 24 h (bearing pMami_*k*) or without induction (bearing pMhsp_*k*) were separated by centrifugation, disrupted at 10 (× 30 s) impulses on a Sonicator Q500 (Qsonica, Newtown, CT, USA), and then lysates were treated in SDS-PAGE sample buffer at 100 °C. Proteins were separated by SDS-PAGE (10% acrylamide/bis-acrylamide) and stained with Coomassie Blue.

### 2.8. Biotransformation of Steroids

Actinobacteria were grown in 65 mL of MYCB medium in 750 mL shake flasks at 200 rpm at 30 or 35 °C for 48 h. The resulting culture (10 mL) was inoculated into TR3 conversion medium (90 mL) containing (g/L): Tween 80—1, yeast extract—10, soy peptone—5, corn steep solids—5, glycerol—10, K_2_HPO_4_·3H_2_O—10, (NH_4_)_2_SO_4_—1, MgSO_4_·7H_2_O—0.2, FeSO_4_·7H_2_O—0.01, ZnSO_4_·7H_2_O—0.002, pH 7.0—7.2, and incubated under the same conditions for 24 or 36 h. In the case of *N. simplex*, 3-KSD activity was induced with 0.2 g/L of cortisone 21-acetate (AcC). In the case of mycolicibacteria harboring pMVT61 or pMami_*k* plasmids, acetamide was added to a final concentration of 2 g/L simultaneously with the inoculation or after 12 h of incubation to induce the expression of *kstD2*_NS_.

To start bioconversion, hydrocortisone (5 g/L, 13.79 mmol/L) or prednisolone (5 g/L, 13.87 mmol/L) were added to 90 mL of bacterial cultures as an aqueous solution with MCD (10 mL) after 24 h since the start of induction. Phytosterol (5 g/L, 12.06 mmol/L) or 6α-methyl-hydrocortisone (5 g/L, 13.28 mmol/L) were added to the transformation medium before sterilization in the form of dry powders. The molar ratio of MCD:substrate was 1.8:1 for cortisol and phytosterol, or 1.9:1 for 6α-methyl-hydrocortisone. At the start of the conversion, the volume of the medium with all additives was 100 mL. Biotransformation was carried out at the same temperature as the cultivation.

In some experiments, 0.1 mM menadione was added to the conversion medium as EEA [42,43], in the form of suspension in methanol (1.25 mL per 100 mL of the medium).

*M. neoaurum* NRRL B-3805∆*kstD* and *M. smegmatis* BD strains carrying pMVT61 or pMV261-N plasmids were used as negative controls.

### 2.9. Steroid Assays

Every 12–24 h of incubation, the evaporation of water from conversion mixtures was controlled gravimetrically and compensated by adding distilled water. Then, samples were taken, extracted with 5 volumes of ethyl acetate for TLC, or diluted 25 times with 50% aqueous acetonitrile for HPLC. The diluted samples were purified by centrifugation (15 min at 6000× *g*).

TLC was carried out on Alugram Sil G/UV254 plates (Macherey-Nagel, Düren, Germany) in benzene:acetone (3:2 *v*/*v*). Steroids with 3-keto-4-en-configuration were evaluated under UV light at 254 nm. For other possible products and sterols, the plates were stained with MnCl_2_-reagent [44] and observed at 365 nm.

HPLC analysis of phytosterol bioconversion products was performed on ODC columns with linear gradient elution as previously reported [45]. For 6α-methyl-hydrocortisone and derivatives, an isocratic elution with mobile phase acetonitrile:water:acetic acid (40:60:0.01, *v*/*v*/*v*) at 50 °C was applied. Hydrocortisone and products of its bioconversion were analyzed according to European Pharmacopoeia [46]. The HPLC signals were calibrated with external standards. In the case of some minor by-products, homologous steroids with 3-keto-4-ene- or 3-keto-1,4-diene chromophores were used for calibration.

### 2.10. Calculations

The specific 3-KSD or 20β-HSD activities of bacterial cells were calculated based on the increment of the concentration of all 1(2)-dehydrogenated or all C20β-reduced products, respectively, between adjacent experimental points (6–24 h) as follows:

(1) $$A=\frac{{∆C}_{Steroids}}{∆t\cdot DCW},$$

**Theorem** **1.** *The specific steroid-1(2)-dehydrogenation activity of bacterial cultures. *A*–specific activity (μmol/(h g));* ${∆C}_{Steroids}$*—increment of concentrations of all 1(2)-dehydro-steroids (μM) for the time period; *∆t—*time period (h);* DCW*—dry cell weight (g/L).*

The molar yields were calculated according to the formula:

(2) $$Y=\frac{\sum_{1}^{n}C_{Pi}}{C_{Substrate}}\times 100\%$$

**Theorem** **2.** *The molar yields.* Y*—molar yield of individual steroid product or series of the steroid products bearing the specific moiety e.g., 1(2)-dehydro- or 20β-hydroxy-(%); *$C_{Pi}$*—concentration and *n *the number of individual steroid products with specific moiety (mM);* $C_{Substrate}$*—the charge of the bioconversion substrate (mmol/L).*

In the material balance of bioconversion, the sum of all 3-keto-4-en- and 3-keto-1,4-dien-steroids detected at UV 254 nm was taken into account. The level of steroid destruction was estimated according to the formula:

(3) $$DF=\frac{C_{Substrate}-\sum_{1}^{n}C_{Pi}}{C_{Substrate}}\times 100\%$$

**Theorem** **3.** *The material balance of bioconversion.* DF*—the fraction of degraded steroids (%, mol/mol);* $C_{Pi}$*—concentration and *n *the total number of individual steroid products detected (mmol);* $C_{Substrate}$*—the initial concentration of the bioconversion substrate (mmol/L).*

### 2.11. Statistics

The experiments were carried out in not less than three repeats. The arithmetic mean values and the corresponding standard errors are presented.

## 3. Results

### 3.1. Construction of Recombinant Mycolicibacterium Strains

For the heterologous expression, the kstD2_NS_ (KR76_27125; GenBank: AIY19529.1) gene was chosen from five genes of 3-KSDs present in the N. simplex VKM Ac-2033D genome. As shown previously, N. simplex exhibits low 3-ketosteroid-1,2-dehydrogenase activity without induction. The expression of kstD2_NS_ was upregulated to the greatest extent in the presence of AcC, while the expression of other paralogs changed insignificantly in the presence of AcC or sterols, which indicates the predominant role of KstD2_NS_ in the dehydrogenation of steroid substrates in N. simplex [16].

As model recipient organisms having endogenous steroid catabolism, the two strains M. neoaurum NRRL B-3805∆kstD [38] and M. smegmatis BD [39] were applied. These strains convert sterols into 1(2)-saturated 3-keto-4-en-steroids—androst-4-en-3,17-dione (AD) and 20-hydroxymethyl-pregn-1-en-3-one (HMP). For both strains, the ability to reduce the 20-carbonyl group of steroids has not been previously described.

A set of recombinant plasmids containing kstD2_NS_ was constructed for expression of the gene in mycolicibacteria under the control of inducible acetamidase (pMami_k) or constitutive hsp60 (pSM_k and pMhsp_k) promoters (Table 1, Figure S1).

Unlike the plasmids pMhsp_k (Figures S2a and S3a) and pMami_k (Figure S3a,b), the plasmid pSM_k was found to be structurally unstable in Mycolicibacterium cells. All the analyzed plasmids isolated from 40 Hyg^R^-transformants of M. smegmatis BD and 38 Hyg^R^-transformants of M. neoaurum obtained after transformation by pSM_k plasmid DNA contained deletions of various lengths (Figure S2b,c). Structural instability of plasmids derived from pSMT3 and containing cloned ORFs under the control of a strong constitutive hsp60 promoter was previously demonstrated in M. tuberculosis, M. bovis, and M. smegmatis cells. It was shown that the deletions affected both the promoter and the coding region of the genes, while, apparently, the stability of the plasmids depended on the sequence of the inserted ORF [40,47,48].

Expression of the kstD2_NS_ gene in M. neoaurum NRRL B-3805∆kstD and M. smegmatis BD cells carrying plasmids pMhsp_k and pMami_k was confirmed by analysis of cell lysates with SDS-PAGE (Figure S4). The analysis revealed an additional protein band of approximately 58 kDa, corresponding to the calculated M_r_ of a KstD2_NS_ protein.

Multiple alignments of the nucleotide sequence derived for the hsp60 promoter from the constructed vector pMV261-N with those previously published for the same locus of the plasmid pMV261 [41], the hsp60 sequence presented by Sun et al., 2020 [49], and the original sequence from Mycobacterium bovis BCG Pasteur 1173P2 [GenBank: AM408590] revealed some variability (Figure S5). Most importantly, the fragment from pMV261-N revealed a deletion of three nucleotides at positions 21–23 from the beginning of the XbaI site, which, however, did not result in a loss of hsp60 promoter functionality.

### 3.2. Biotransformation of Steroids by Recombinant Strains of M. neoaurum B-3805∆kstD

**Figure 2:**
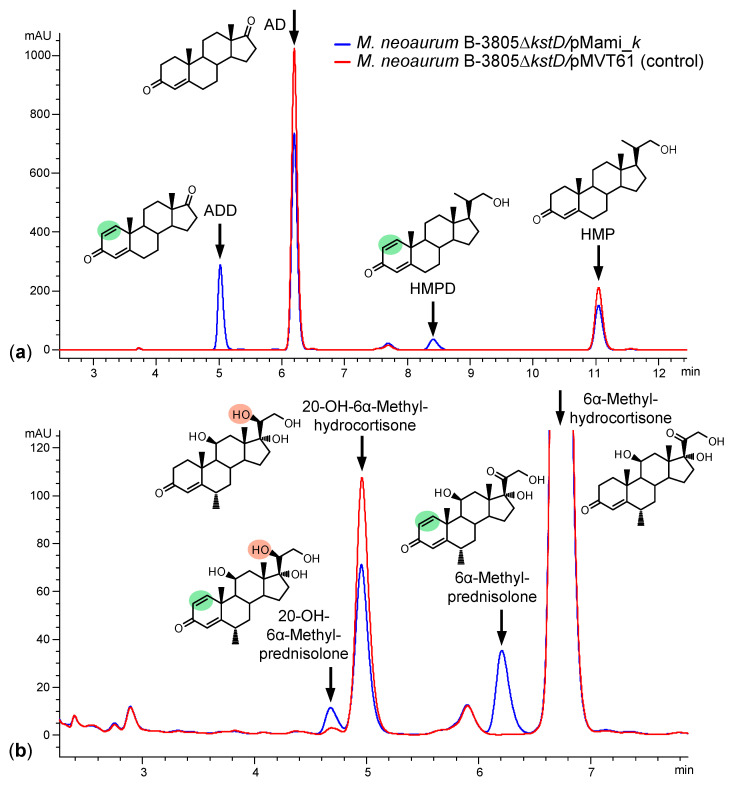
HPLC profiles of the biotransformations of phytosterol (**a**) and 6α-methyl-hydrocortisone (**b**) with M. neoaurum B-3805∆kstD/pMami_k and the control strain B-3805∆kstD/pMVT61. AD—androst-4-ene-3,17-dione, ADD—androsta-1,4-diene-3,17-dione, HMP—20-hydroxymethylpregn-4-en-3-one, HMPD—20-hydroxymethylpregna-1,4-dien-3-one. Green area—1(2)-double bond, orange area—20β-hydroxy group.

The ability of the obtained strain M. neoaurum B-3805∆kstD/pMami_k to produce 1(2)-dehydrogenated 3-ketosteroids was analyzed upon biotransformation of three bioconversion substrates. Bioconversion of phytosterol (Figure 2a) and 6α-methyl-hydrocortisone (Figure 2b) was carried out by growing M. neoaurum B-3805∆kstD/pMami_k cells in the presence of acetamide. In the negative control, the strain M. neoaurum B-3805∆kstD/pMVT61 (without kstD2_NS_ insert) as well as the plasmid-free parent strain B-3805∆kstD (not shown) converted phytosterol exclusively into 1(2)-saturated products. At the same time, B-3805∆kstD/pMami_k, along with AD and HMP, produced the corresponding 1(2)-dehydroanalogues (ADD and HMPD) with a total molar yield of 24.3% (Figure 2a). The cells of B-3805∆kstD/pMami_k showed lower steroid-1(2)-dehydrogenase activity against 6α-methyl-hydrocortisone, thus providing the molar yield of 6α-methyl-prednisolone of no more than 6% after 120 h incubation (Figure 2b).

**Figure 3:**
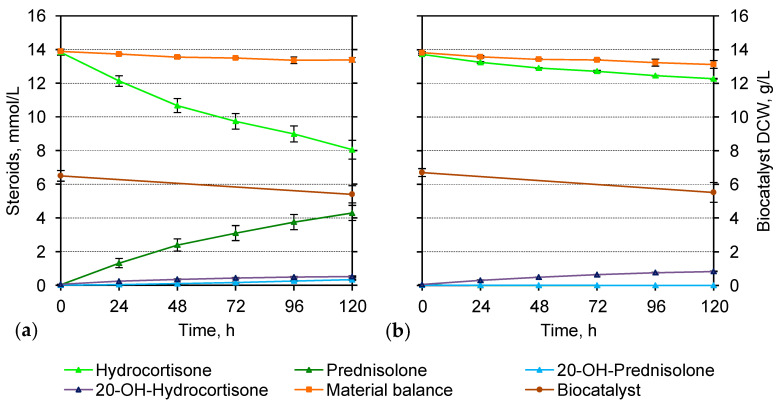
Time course of hydrocortisone biotransformation (**a**) by the growing cells of M. neoaurum B-3805∆kstD/pMami_k expressing kstD2_NS_; (**b**) in the negative control (B-3805∆kstD/pMVT61). The cells were cultured in TR3 medium for 36 h including 24 h acetamide induction at 30 °C, and then hydrocortisone (13.79 mmol/L) was added. 20-OH-Hydrocortisone—11β,17α,20β,21-tetrahydroxypregn-4-ene-3-one, 20-OH-Prednisolone—11β,17α,20β,21-tetrahydroxypregna-1,4-diene-3-one.

For the biotransformation of hydrocortisone, the cultures at the end of the active growth phase (24 or 36 h) were induced for 24 h before the substrate addition. The growth of the experimental and control cultures did not differ significantly and did not depend on the presence of acetamide. The culture densities at the time of hydrocortisone addition were approximately 6.3 g/L (DCW) and slightly decreased over the bioconversion period (Figure 3a,b).

The maximum specific 3-KSD activity of M. neoaurum B-3805∆kstD/pMami_k cells towards hydrocortisone (13.79 mmol/L) was of 9.75 ± 1.83 µmol/(h × g) (DCW) (Table 2). Prednisolone accumulated as the main biotransformation product with a molar yield of 32.74 ± 3.26% after 120 h (Table 2). The conversion rate was the highest in the first 24 h, and then steadily decreased, while a significant part of the substrate remained unconverted (Figure 3a).

The control strain *M. neoaurum* B-3805∆*kstD/*pMVT61 produced 20β-reduced hydrocortisone (11β,17α,20β,21-tetrahydroxypregn-4-en-3-one), and *M. neoaurum* B-3805∆*kstD*/pMami_*k* also produced the corresponding 1(2)-dehydroanalogue (11β,17α,20β,21-tetrahydroxypregna-1,4-diene-3-one), indicating the presence of weak endogenous reducing activity towards the 20-carbonyl group of steroid substrates in *M. neoaurum* (Figure 2b).

### 3.3. Biotransformation of Hydrocortisone by Recombinant Strains of M. smegmatis

**Figure 4:**
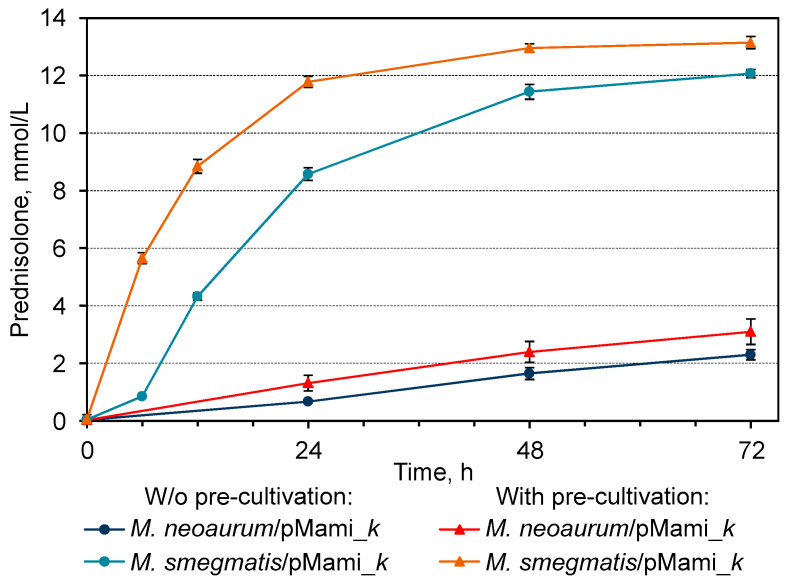
Prednisolone accumulation curves by recombinant cells of *M. neoaurum* B-3805∆*kstD/*pMami_*k* and *M. smegmatis* BD*/*pMami_*k* induced by acetamide with or without 12 h pre-cultivation. The cells were cultured in TR3 medium for 24 h in the presence of acetamide or during 36 h with 12 h pre-cultultivation followed by 24 h induction, and then hydrocortisone (13.79 mmol/L) was added.

Growing cells of *M. smegmatis* BD/pMami_*k*, engineered from an alternative host strain but expressing *kstD2*_NS_ under the control of the same inducible acetamidase promoter, exhibited 10–11 times higher steroid-1(2)-dehydrogenase activity towards hydrocortisone compared to *M. neoaurum* B-3805∆*kstD*/pMami_*k* (Table 2, Figure 4). The cells of *M. smegmatis* BD in negative controls showed insignificant endogenous 3-ketosteroid-1(2)-dehydrogenase activity against hydrocortisone 0.3–0.7 µmol/(h (× g)) (DCW) (Table 2).

The steroid-1(2)-dehydrogenase activity of *M. smegmatis* BD/pMami_*k* depended on the conditions of the acetamide induction of *kstD2*_NS_ expression. A prolongation of overall cultivation period from 24 to 36 h with the addition of acetamide after 12 h pre-cultivation provided complete conversion of the substrate and resulted in 1.5-fold increase of the maximal specific activity as compared with the shorter cultivation time (24 h) and earlier addition of acetamide (0 h) (Table 2). In addition, the pre-cultivation positively effected on the bioconversion dynamics: the maximum activity was observed immediately after addition of hydrocortisone, while in 24 h-old cells it occurred from 6 to 12 h of bioconversion (Figure 4).

**Figure 5:**
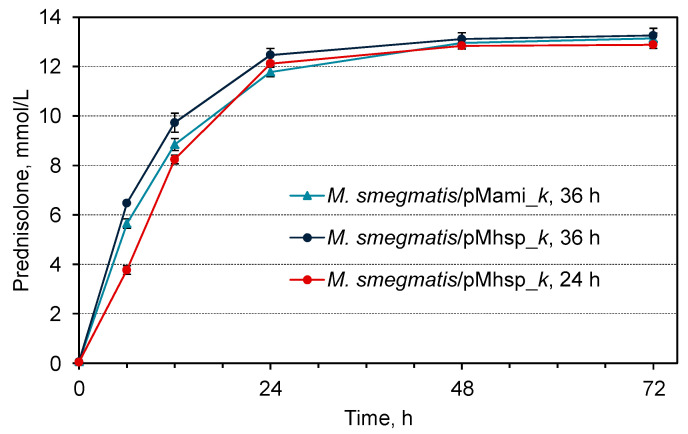
Prednisolone accumulation curves at hydrocortisone (13.79 mmol/L) bioconversion by cells of *M. smegmatis* BD*/*pMhsp_*k* grown for 24 h or 36 h, and by cells of *M. smegmatis* BD*/*pMami_*k* grown for 36 h (including 12 h pre-cultivation and 24-h acetamide-induction).

The maximum specific 3-KSD activity was 1.2 times higher in *M. smegmatis* BD/pMhsp_*k* cells expressing *kstD2*_NS_ under the control of the constitutive *hsp60* promoter (Table 2). At the same time, the dynamics of prednisolone production in the case of the *hsp60* promoter were similar to those for the acetamidase promoter under the best induction conditions at 30 °C (Figure 5).

The use of an older *M. smegmatis* BD/pMhsp_*k* culture grown for 36 h effected an increase in the maximum KstD*2*_NS_ activity compared to 24 h culture; the highest activity level (123.2 ± 3.67 µmol/(h (× g)) (DCW) was observed in the period from 0 to 6 h bioconversion (Table 2). Simultaneously, with the use of the *hsp60* promoter, the dynamics of accumulation and final yield of prednisolone were less sensitive to the cultivation duration as in the case of the acetamidase promoter (Figure 5).

Under the optimal biotransformation conditions with *M. smegmatis* BD/pMhsp_*k*, the complete conversion of the substrate was observed after 48 h, thus resulting in the highest cumulative molar yield of 1(2)-dehydrogenated products (96.7 ± 1.12%) (Table 2).

At the incubation of control or *kstD2*_NS_-expressing *M. smegmatis* BD cells with hydrocortisone, the accumulation of C20β-reduced products was observed similarly to that in *M. neoaurum* B-3805∆*kstD* strains (Table 2, Figure S6).

The densities of the 24 h-old cultures of *M. smegmatis* BD strains bearing different plasmids and grown at 30 °C with or without acetamide varied insignificantly at the bioconversion start (8.21 ± 0.1 g/L (DCW)), while at 35 °C they were noticeably lower (6.49 ± 0.23 g/L (DCW)). After 36 h cultivation at 30 °C, the cultures were slightly denser (8.94 ± 0.16 g/L (DCW)). During the bioconversion, the culture densities decreased by 5–15% (Figure S6).

### 3.4. Steroid 20-Carbonyl Group Reduction

**Figure 6:**
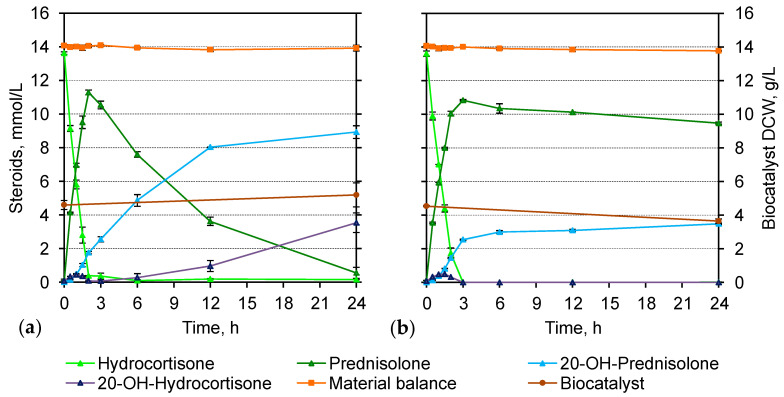
Time course of hydrocortisone biotransformation (13.79 mmol/L) by growing cells of *N. simplex* VKM Ac-2033D. The cells were cultivated and induced with AcC for 24 h at 30 °C: (**a**) without menadione; (**b**) in the presence of 0.1 mM menadione.

As follows from Table 2, the maximum specific 3-KSD activity of the AcC-induced *N. simplex* cells was significantly (15–18 times) higher than that of *kstD2*_NS_-expressing *Mycolicibacterium* strains. Hydrocortisone was fully converted by *N. simplex* cells within 3 h with a cumulative molar yield of 1(2)-dehydro-steroids (97.6 ± 1.39%) (Figure 6a). Meanwhile, *N. simplex* cells showed high reduction activity of the 20-carbonyl group of steroids (Table S2), which led to the accumulation of 20.7 ± 1.03% of 20-OH-prednisolone and 20-OH-hydrocortisone, thus resulting in a decrease in prednisolone yield (Table 2). Application of EEA menadione only partly inhibited this undesirable activity (Figure 6b).

The level of steroid 20-carbonyl reduction activity in *Mycolicibacterium* cells was noticeably lower than in *N. simplex* (130–660 times, Table S2). Both strains of *M. neoaurum* during the biotransformation of hydrocortisone produced about 6% (mol/mol) of 20β-reduced steroids (mainly 20-OH-prednisolone) for 120 h, while *M. smegmatis* BD strains produced even less 20β-reduced steroids (0.7–4.0%) 20β-reduced steroids for 48 h depending on the cultivation and bioconversion conditions (Table 2).

**Figure 7:**
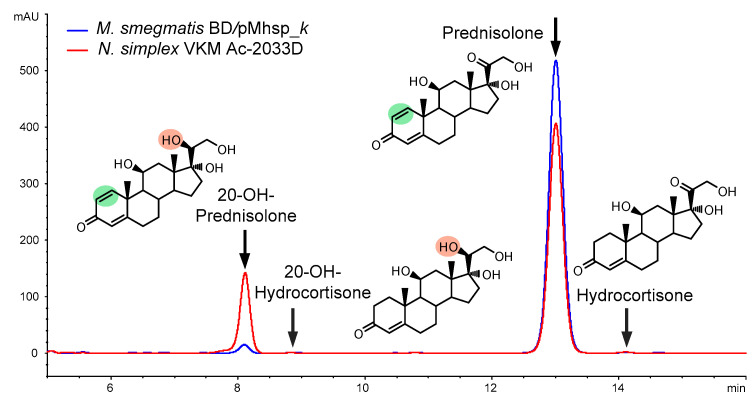
Comparison of HPLC profiles after complete hydrocortisone conversion (13.79 mmol/L) by *N. simplex* cells (conversion time 3 h) and *M. smegmatis* BD/pMhsp_*k* cells (conversion time 48 h). Green area—1(2)-double bond, orange area—20β-hydroxy group.

The comparison of steroid profiles obtained under the optimized conditions of hydrocortisone biotransformation by *N. simplex* and recombinant *M. smegmatis* BD/pMhsp_*k* showed better selectivity for the latter (Figure 7). Despite a lower level of the target 3-KSD activity, *M. smegmatis* BD/pMhsp_*k* provided a higher molar yield of prednisolone (95.43 ± 1.72% vs. 77.27 ± 2.18%) (Table 2).

### 3.5. Estimation of Minor Modifications and Destruction of Steroids

During the bioconversion of hydrocortisone, a decrease in the estimated total content of 3-keto-4-ene-steroids (Figure 3a and Figure S6) and the appearance of trace signals of 10–12 products were observed, in addition to those indicated in Figure 1. The trace products presumably were steroids with the 3-keto-4-ene- or 3-keto-1,4-diene-configuration of the steroid core, as evidenced by the high absorbance at 254 nm. Similar trace signals were also observed in the case of *N. simplex*. In addition, mycolicibacteria (but not *N. simplex*) produced three trace products that did not absorb UV at 254 nm and were detected on TLC after staining with MnCl_2_-reagent (Figure S7). Despite the presence of trace by-products, a rough estimate of the material conversion balance suggests that a small part of the substrate has undergone irreversible degradation. The fraction of steroids involved in degradation in *M. neoaurum* B-3805∆*kstD* strains varied from 4.65 ± 0.76 to 9.51 ± 0.82% (after 120 h) and from 0.43 ± 0.03% to 6.69 ± 1.44% in *M. smegmatis* BD strains (after 48 h) depending on the incubation conditions. *N. simplex* degraded 0.46 ± 0.039% of hydrocortisone for 3 h of bioconversion (Table 2, Figure S6).

### 3.6. Reverse Activity of 1(2)-Hydrogenation of Prednisolone

As follows from Figure 6a, the continued incubation of *N. simplex* cells in the bioconversion mixture with prednisolone and 20-OH-prednisolone after the depletion of the initial substrate (hydrocortisone) resulted in the accumulation of approximately 25% (mol/mol) of 20-OH-hydrocortisone for 21 h. This is possible due to the reverse activity of 1(2)-hydrogenation of the accumulated steroid products. The activity was eliminated with menadione (Figure 6b).

In the recombinant *Mycolicibacterium* cells bearing the control plasmids without *kstD2*_NS_ insert, the activity of 1(2)-hydrogenation of prednisolone (13.87 mmol/L) was 2–3 orders of magnitude lower than the levels of 3-KSD activity in the *kstD2*_NS_-expressing cells. Moreover, the 1(2)-hydrogenation activity did not change with variations in the shaking frequency of the flasks in the range of 100–200 rpm and did not depend on the presence of acetamide (Table S3).

### 3.7. The Effect of Temperature on Hydrocortisone Bioconversion by Mycolicibacteria

**Figure 8:**
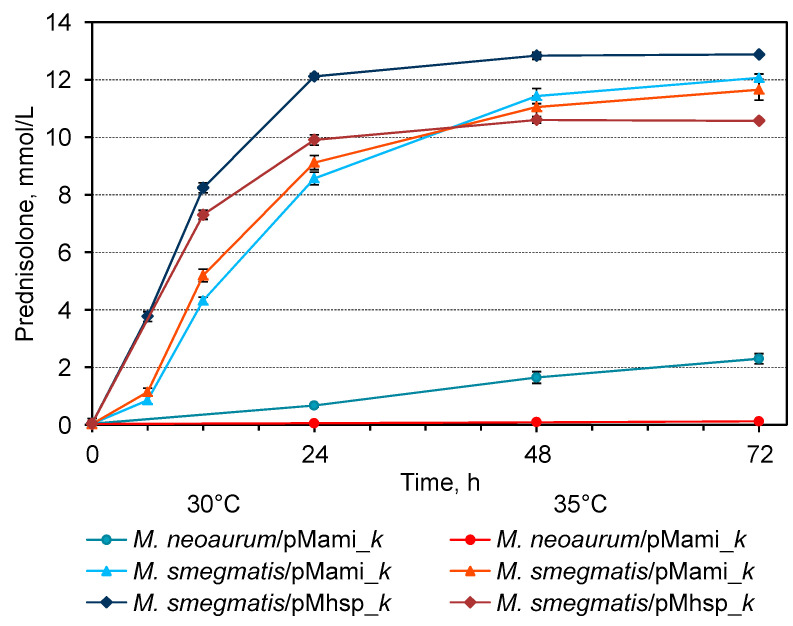
Prednisolone accumulation curves at hydrocortisone (13.79 mmol/L) bioconversion by recombinant cells of *M. neoaurum* B-3805∆*kstD/*pMami_*k*, *M. smegmatis* BD*/*pMami_*k*, and *M. smegmatis* BD*/*pMhsp_*k* at 30 °C or 35 °C The cells were cultured in TR3 medium for 24 h at the same temperatures as for the bioconversion (cells bearing the pMami_*k* plasmid were grown in the presence of acetamide).

For all the recombinant strains, the increase in temperature from 30 °C to 35 °C did not significantly affect the growth at the stage of submerged cultivation (Figure S6). At the same time, with an increase in cultivation and bioconversion temperature from 30 °C to 35 °C, the specific activity of KstD2_NS_ drastically decreased (approximately 23 times) in the cells of *M. neoaurum* B-3805∆*kstD*/pMami_*k*, while in the cells of *M. smegmatis*, on the contrary, it increased by 1.5 times (BD/pMami_*k*) or remained unchanged (BD/pMhsp_*k*) (Table 2, Figure 8).

In all *Mycolicibacterium* strains, the rise in bioconversion temperature resulted in an increase in the yield of C20β-reduced products (in 1.23–2.74 times) and the fraction of steroids involved in destruction, e.g., in 1.34–2.09 times in *M. smegmatis* BD/pMami_*k* (Table 2). The estimation of the maximum specific activity of the undesirable steroid C20β-reduction at different incubation temperatures is presented in Table S2.

## 4. Discussion

Despite significant progress in the field of microbial production of 1-dehydroanalogues of steroids, a number of problems remain unresolved. First, this concerns the presence of undesirable side activities in industrial biocatalyst strains, leading to the reduction of the 20-keto group and the 1(2)-double bond. In this study, these activities were investigated in more detail for the industrial strain *N. simplex* VKM Ac-2033D. The strain produced up to 20% of steroid 20β-alcohols and reduced the 1(2)-double bond of prednisolone to form the corresponding 1(2)-saturated steroid. Our attempts to block these undesirable activities in *N. simplex* failed because of the absence of the corresponding genetic tools and the instability of the plasmids introduced in this actinobacteria. Notably, publications on genetic manipulations with relative actinobacteria (e.g., *Pimelobacter simplex*, *Arthrobacter simplex*, etc.) are scarce.

To solve the problem of selectivity of steroid 1(2)-dehydrogenation by whole-cell catalysis, we studied the heterologous expression of the gene coding for KstD2_NS_, whose expression was found to be the most highly upregulated among the five paralogs presented in the *N. simplex* genome. In contrast to the known studies on the heterologous expression of 3-KSDs in steroid-oxidizing bacteria [34,35], we chose as recipients the strains of mycolicibacteria lacking endogenous 3-KSD activity. This made it possible to compare the in vivo activity of heterologous 3-KSD in the hosts of two *Mycolicibacterium* species.

The expression of *kstD2*_NS_ in *M. neoaurum* B-3805∆*kstD* did not affect the ability of bacteria to convert phytosterol, but the activity of KstD2_NS_ towards sterol catabolism intermediates was insignificant. Closely related wild-type strains with their own 3-KSDs, such as *M. neoaurum* VKM Ac-1816D [50] and *M. neoaurum* JC-12 [51], produced under similar conditions more than 90% of 1(2)-dehydrogenated products in total.

The recombinant *M. neoaurum* B-3805∆*kstD*/pMami_*k* 1(2)-dehydrogenated exogenous pregnane steroid substrates, both natural (hydrocortisone) and synthetic (6α-methyl-hydrocortisone). The activity towards the latter was significantly lower, which correlates with the data obtained earlier for *N. simplex* VKM Ac-2033D [52,53].

Recombinant strains derived from *M. smegmatis* showed a higher target 3-KSD activity towards hydrocortisone than strains based on the *M. neoaurum* host. Both parent strains (*M. smegmatis* BD and *M. neoaurum* B-3805∆*kstD*) and their recombinants showed some 20β-reductase activity, but its level was significantly (in hundreds of times) lower as compared with that of growing *N. simplex* cells (Table S2) and was almost the same as shown earlier for the resting *N. simplex* [54].

Another undesirable activity that may accompany target 1(2)-dehydrogenation during a whole-cell biocatalysis is reverse reaction, —reduction of the C1-C2-double bond to form the corresponding 1(2)-saturated steroids. This activity was firstly demonstrated in *Mycolicibacterium* sp. NRRL 3805 (later re-classified as *Mycolicibacterium neoaurum*) [55] and the corresponding enzyme was characterized as NADP(H)-dependent reductase [19,55]. However, since that time, no one has reported the gene coding for this reductase, and there is a discussion in the literature whether the same enzyme catalyze the 1(2)-dehydrogenation and the reverse reaction or not. In this study, we observed a very low level of endogenous steroid-1(2)-reducing activity toward prednisolone in *Mycolicibacterium* cells in the conditions applied. The presence of the NAD(H)-dependent 1-reductase activity towards ADD in two *Mycolicibacterium* strains has been recently demonstrated in vitro and in vivo [56]. The presence of 1-reductase activity in *N. simplex* has been evidenced earlier [11,57] and was confirmed in this study. Noteworthy, no 1(2)-hydrogenation was observed in *N. simplex* in the presence of EEA menadione.

Menadione is able to bypass the electron transport chain of *N. simplex* from organic substrates to oxygen at the level of FAD—menaquinone, creating a deficiency of reduced cofactors (NADH) in the cell and intensifying the 1,2-dehydrogenation of steroids. Optimal concentrations of menadione were previously empirically selected for resting and starving *N. simplex* cells and made it possible to effectively prevent the residual activity of reducing the 20-carbonyl group of pregnane substrates [42,43]. In our experiments, growing *N. simplex* cells appeared to be able to compensate for the deficiency of reduced equivalents from endogenous or exogenous carbon sources, which promoted the reduction of the 20-carbonyl group of hydrocortisone and prednisolone (presumably an NADH-dependent process). At the same time, menadione hindered the reverse transfer of electrons from FAD to the steroid molecule, thereby preventing 1,2-hydrogenation.

As reported earlier [34], expression of additional genes coding for 3-KSD under *hsp60* control in *M. neoaurum* NwIB-01 resulted in an increased yield of ADD from AD (0.4 g/L). It is noteworthy that the introduction of an additional copy of the homologous KstD_M_ gene increased the selectivity of ADD production to a greater extent (from 68.9% to 98.3–98.6%) than the introduction of an additional heterologous KstD_A_ gene from *A. simplex* 156 (up to 86.5–91.1%). The data presented in this work allow us to estimate approximately the level of activity of additional 3-KSDs as an order of magnitude lower than that obtained by us for *M. neoaurum* B-3805∆*kstD*/pMami_*k.*

At the same time, in the most successful studies on the expression of genes of heterologous 3-KSD in microbial hosts that do not possess endogenous sterol catabolism systems, significantly higher activities were demonstrated during the conversion of steroids by washed recombinant cells. Thus, the specific productivity at the conversion of hydrocortisone to prednisolone by *E. coli* BL21 cells expressing the synthetic *prkstD* gene [30] and the conversion of AD to ADD by *B. subtilis* cells expressing the codon-optimized *kstD* gene from *M. neoaurum* JC-12 [31] were approximately 30–40 times higher than the activity obtained in this study for *M. smegmatis* BD/pMhsp_*k*.

Possible reasons for the lower activity of KstD2_NS_ observed in our study may be a deficiency in the cells of mycolicibacteria of the necessary redox partners and insufficient level of the gene expression, or transport limitations through the cell wall for steroids. It should be noted that the level of KstD2_NS_ activity in vivo was significantly higher in the recombinant strains derived from *M. smegmatis* mc^2^ 155. This strain has a cell wall defect (mycolic acid deficiency) [58,59], which can presumably intensify the passive transport of hydrophobic steroid compounds. It was previously shown that the disruption of the proportion of mycolic acids in *M. neoaurum* ATCC 25795 due to the knockout of the *kasB* gene encoding β-ketoacyl carrier synthetase led to an increase in cell wall permeability for hydrophobic dyes and a 2.38-fold increase in the production of 9α-hydroxyandrostenedione from phytosterol [60]. The endogenous reverse activity of C1(2)-reduction in *Mycolicibacterium* strains observed in our work was negligible and cannot be the reason for the low apparent activity of KstD2_NS_.

The acetamidase and *hsp*60 promoters used in this work were previously characterized by the expression of various genes in *M. smegmatis* as being among the strongest. When the genes coding for marker fluorescent proteins and the genes *kshA* and *kshB* encoding 3-ketosteroid-9α-hydroxylase were expressed in *M. smegmatis*, the *hsp*60 promoter was inferior in strength only to the artificial CP6 promoter [49]. Previously, the inducible acetamidase promoter from *M. smegmatis* mc^2^ 155 [61] was successfully used for heterologous expression of genes of steroidogenesis in closely related strains [39]. In the current work, a direct comparison of the strengths of the acetamidase and *hsp*60 promoters was performed for the first time. The level of 3-KSD activity in recombinant *M. smegmatis* expressing *kstD2*_NS_ under their control was comparable but slightly higher in the case of *hsp60* (Table 2, Figure 5). In practice, the acetamidase promoter is less convenient since the induction of expression requires the introduction of a significant amount of acetamide, which can change the carbon-to-nitrogen ratio. Also, the hydrolysis of acetamide by bacterial cells can lead to alkalization of the medium.

An important characteristic of potential producer strains is the level of undesirable degradation activity of steroid substrates. All studied strains under the described conditions had a limited ability to degrade hydrocortisone and convert it to a number of trace products. The maximum lack in material balance over the entire period of active conversion (until the maximum yield of prednisolone was reached) was observed at 35 °C and comprised 6.69 ± 1.44% for *M. smegmatis* BD/pMV261-N (after 48 h) and 9.51 ± 0.82% for *M. neoaurum* B-3805∆*kstD*/pMVT61 (after 120 h). The supposed steroid degradation by *M. smegmatis* BD strains decreased when the temperature was lowered to 30 °C (by 1.7–2.1 times) and with the use of more aged cells (by 2.7–5.2 times). The smallest amount of steroids involved in degradation during the period of active conversion (less than 0.5%) was observed in *N. simplex* and *M. smegmatis* BD/pMhsp_*k* (Table 2).

The parent strain *M. neoaurum* B-3805∆*kstD* [38] used in this work was constructed on the basis of a known industrial AD producer obtained by statistical mutagenesis and showing no activity of 3-ketosteroid-9α-hydroxylase [62]. The strain *M. smegmatis* BD was obtained from *M. smegmatis* mc^2^ 155 [63] by gene knockout of the reductase subunit of 3-ketosteroid-9α-hydroxylase *kshB* (*MSMEG_6039_kshB*) and the *kstD* gene (*MSMEG_5941_kstD*) [39]. Thus, in both parental strains, in contrast to *N. simplex*, a set of genes encoding key enzymes of steroid core degradation were inactivated [8]. Also, the ability to degrade sterol biotransformation products was not previously shown for them. In this work, in *M. smegmatis* BD cells, but not in *M. neoaurum* B-3805∆*kstD* cells, a slight 3-ketosteroid-1(2)-dehydrogenase activity was observed, apparently associated with the functioning of “minor” 3-KSD encoded by the *kstD2* and *kstD3* genes, and noticed in some mycolicibacteria [64].

The degradation of pregnane steroids by actinobacteria has not been sufficiently studied. The complete degradation of hydrocortisone by *Rhodococcus zopfii* without accumulation of intermediate products has been described, which presumably proceeds through 9-hydroxy-11-keto-androstadienedione [65] with the opening of the steroid ring B. The *Mycolicibacterium* strains used in this work did not show the activity of 3-keto-steroid 9α-hydroxylase (KSH), which is necessary for opening the steroid ring B along with 3-KSD [38,39]. Along with *kstD* (*MSMEG_5941_kstD*) inactivated in the BD strain, there are three putative “minor” 3-KSD genes (*MSMEG_2867*, *MSMEG_2869*, and *MSMEG_4864*) in the *M. smegmatis* mc^2^ 155 genome, whose level of expression slightly increases in response to 3-keto-steroids and cholesterol [64]. At the same time, activation of “minor” endogenous 3-KSD cannot lead by itself to the destruction of hydrocortisone but should increase the steroid-1(2)-dehydrogenase activity of control cells, which was not observed in the experiments.

The significant level of hydrocortisone degradation observed in some cases may be associated with the functioning of “silent pathways” of steroid oxidation, such as the C-19+ catabolic pathway found in *M. smegmatis* mc^2^ 155 [66]. Expression of the genes of the C-19+ pathway, located in the gene cluster of the same name, is tightly regulated and is not activated in the presence of phytosterol or C19-steroids. Despite the fact that the natural mechanisms of activation of the C-19+ pathway have not been established, it can be assumed that the expression of the genes of this cluster or a putative unknown operon containing minor genes for steroid catabolism was activated at incubation of mycolicybacteria in a nutrient medium with hydrocortisone and its conversion products at elevated temperature.

The known temperature optima for both the growth and steroid-transforming activity of *N. simplex* VKM Ac-2033D or related strain *A. simplex* 156 are 30 or 32 °C, respectively [9,35]. As shown earlier, the temperature optima for the activity of heterologously expressed 3-KSDs from different mesophilic strains were within 30–35 °C [23,29,30,31,32] with the exception of 3-KSD from *Mycobacterium neoaurum* DSM 1381 expressed in *E. coli* (40 °C) [26]. Meanwhile, activity of two KstDs (KstD2, KstD3) from *M. neoaurum* NwIB-R10hsd4A expressed in *E. coli* BL21(DE3) was inhibited with an increase in temperature from 30 to 37 °C [67]. In our study, the low KstD2_NS_ activity in *M. neoaurum*/pMami_*k* cells observed at 30 °C decreased to an almost negligible level with an increase in temperature of only 5 °C. On the contrary, while using *M. smegmatis* BD as the host strain, the activity of KstD2_NS_ in vivo was slightly higher at 35 °C, which correlated with the higher temperature optimum of the host strain.

## 5. Conclusions

The heterologous expression of 3-KSD encoded by *kstD2*_NS_ from *Nocardioides simplex* VKM Ac-2033D was carried out in two *Mycolicibacterium* strains under the control of two strong promoters and analyzed based on the target activity of KstD2_NS_ in model whole-cell bioconversions. It was found that KstD2_NS_ was functional in mycolicibacteria; however, the specific activity towards hydrocortisone was noticeably higher when using *M. smegmatis* BD compared to *M. neoaurum* as a host strain.

The strengths of the inducible acetamidase and constitutive *hsp60* promoters in *M. smegmatis* strains expressing *kstD*_NS_ were found to be similar, but in the case of *hsp60*, slightly higher target activity was noted against the background of greater ease of operation. The strain *M. smegmatis* BD in combination with constructed pMami_*k* and pMhsp_*k* recombinant plasmids turned out to be convenient tools for in vivo estimation of the individual activity of the heterologous 3-KSD.

The high selectivity of *M. smegmatis* BD/pMhsp_*k* as a biocatalyst was demonstrated, thus indicating the promise of a technologically simple approach for 1(2)-dehydrogenation of steroids with growing actinobacterial cells expressing heterologous genes of 3-KSD.

Further research will be aimed at increasing recombinant biocatalyst productivity using mycolicibacterial hosts with active intrinsic 3-KSD and heterologous expression of *kstD*s from other sources.

## Figures and Tables

**Table 2:** Steroid-1(2)-dehydrogenating activity and the side activities of the growing actinobacterial cells expressing kstD2_NS_ and the control cells towards hydrocortisone *.

Strain	Cultivation and Bioconversion Conditions			Maximal Specific Steroid 1(2)-Dehydrogenase Activity, μmol/(h × g) (DCW)	Molar Yield, % (mol/mol)			Estimation of Steroid Destruction, % (mol/mol)
					1(2)-Dehydro-steroids	Prednisolone	20β-Hydroxy-steroids	
	Growth Duration before Addition of Hydrocortisone, h	t, °C	Bioconversion Duration, h					
*M. neoaurum* B-3805∆*kstD*/pMami_*k*	24 **	30	120	7.11 ± 1.01	19.81 ± 1.48	18.26 ± 1.12	5.48 ± 0.29	5.02 ± 0.40
	24 **	35	120	0.31 ± 0.01	1.12 ± 0.032	1.12 ± 0.032	6.73 ± 0.44	8.41 ± 0.34
	36 **	30	120	9.75 ± 2.83	35.24 ± 4.1	32.74 ± 3.26	6.24 ± 0.76	5.57 ± 0.86
*M. neoaurum* B-3805∆*kstD*/pMVT61	36 **	30	120	0	0	0	6.11 ± 0.19	4.65 ± 0.75
	24 **	35	120	0	0	0	7.19 ± 1.04	9.51 ± 0.82
*M. smegmatis* BD/pMami_*k*	24 **	30	48	71.19 ± 2.37	82.38 ± 3.54	81.20 ± 2.71	1.34 ± 0.24	4.63 ± 0.92
	24 **	35	48	108.01 ± 5.28	80.74 ± 2.66	78.77 ± 2.73	2.73 ± 0.36	6.23 ± 2.06
	36 **	30	48	102.82 ± 2.14	95.46 ± 1.73	94.14 ± 1.79	1.32 ± 0.21	2.74 ± 0.82
*M. smegmatis* BD/pMVT61	24 **	35	48	0.759 ± 0.023	0.64 ± 0.012	0.64 ± 0.012	4.02 ± 0.18	5.32 ± 0.89
*M. smegmatis* BD/pMhsp_*k*	24	30	48	93.26 ± 2.88	94.63 ± 1.15	93.42 ± 0.88	0.81 ± 0.09	2.16 ± 0.18
	24	35	48	92.82 ± 1.91	88.56 ± 0.28	86.97 ± 0.28	1.67 ± 0.34	4.51 ± 0.27
	36	30	48	123.23 ± 3.67	96.73 ± 1.12	95.43 ± 1.72	0.71 ± 0.12	0.43 ± 0.03
*M. smegmatis* BD/pMV261-N	24	35	48	0.719 ± 0.03	0.69 ± 0.026	0.69 ± 0.026	2.48 ± 0.05	6.69 ± 1.44
	36	30	48	0.329 ± 0.01	0.58 ± 0.042	0.58 ± 0.042	2.55 ± 0.68	5.83 ± 1.61
*N. simplex* VKM Ac-2033D	24 **	30	3	1922.1 ± 41.3	97.63 ± 1.39	77.27 ± 2.18	20.7 ± 1.03	0.46 ± 0.039

*—substrate concentration 13.79 mmol/L; **—including the period of induction (24 h).

## Data Availability

The data presented in this study are available in the article “Reconstruction of steroid 1(2)-dehydrogenation system from *Nocardioides simplex* VKM Ac-2033D in *Mycolicibacterium* hosts” and in the Supplementary Materials, https://www.mdpi.com/article/10.3390/microorganisms11112720/s1.

## Supplementary Materials

The following supporting information can be downloaded: https://www.mdpi.com/article/10.3390/microorganisms11112720/s1, Figure S1: Graphical representation of the construction of recombinant expression plasmids pMhsp_*k*, pMami_*k*, and pSM_*k* containing *kstD2*_NS_; Figure S2: Analysis of plasmid DNA isolated from *Mycolicibacterium* Km^R^-transformants. (a) *Nde*I-site linearized plasmid DNA: from individual Km^R^-clones of *M. smegmatis* BD electroporated with pMhsp_*k* (lanes 1–4); original plasmid pMhsp_*k* from *E. coli* used for electroporation (lane C). (b) *Hin*dIII-site linearized plasmid DNA: pSM_*k* from *E. coli* used for electroporation (lane C); from individual Hyg^R^-clones of *M. smegmatis* BD electroporated with pSM_*k* (lanes 1–6). (c) Native plasmid DNA: pSM_*k* from *E. coli* used for electroporation (C); from individual Hyg^R^-clones of *M. neoaurum* electroporated with pSM_*k* (lanes 1–6). M—DNA ladder (Thermo Fisher Scientific, USA); Figure S3: Confirmation of the presence of the *kstD2*_NS_ gene insert (1.6 kb) by PCR analysis(a) Individual Km^R^-clones of *M. smegmatis* BD bearing pMhsp_*k* (lane 1) or pMami_*k* (lane 2); C—amplicon from the original plasmid pMhsp_*k* isolated from *E. coli*. (b) Individual Km^R^-clones of *M. neoaurum* B-3805∆*kstD*, bearing pMami_*k*. DNA ladder (Thermo Fisher Scientific, USA); Figure S4: SDS-PAGE analysis of KsdD2 (58.5 kDa) in cell-free extracts of recombinant mycolicibacteria: acetamide-induced *M. neoaurum* B-3805∆*kstD*/pMami_*k* (lane 1); negative control—acetamide-induced *M. neoaurum* B-3805∆*kstD*/pMVT61 (lane 2); *M. smegmatis* BD/pMhsp_*k* (lane 3); acetamide-induced *M. smegmatis* BD/pMami_*k* (lane 4); negative control—acetamide-induced *M. smegmatis* BD/pMVT61_*k* (lane 5); M—Protein Ladder (Protein marker (Precision Plus Protein Dual Color Standards, Bio-Rad, USA); Figure S5: Multiple alignment of nucleotide sequences of the *Xba*I-*Hin*dIII fragment from the plasmids pMV261-N used in this work, pMV261 [41], *hsp60* promoter from *M. bovis* BCG Pasteur 1173P2 (GenBank: AM408590.1), and the sequence of *hsp60* promoter reported by Sun et al., 2020 [49]; Figure S6: Dynamics of hydrocortisone biotransformation by the cells of *M. smegmatis* BD and *M. neoaurum* B-3805∆*kstD* bearing experimental plasmids and expressing *kstD2*_NS_ under the control of acetamidase (a–d,h) or *hsp60* (e–g) promoters at 30 °C (a,c,e,g,h) or 35 °C (b,d,f). The cells were cultured in TR3 medium for 24 or 36 h, including 24 h acetamide induction, before the addition of the bioconversion substrate (hydrocortisone, 13.79 mmol/L). 20-OH-Hydrocortisone—11β,17α,20β,21-tetrahydroxypregn-4-ene-3-one. 20-OH-Prednisolone—11β,17α,20β,21-tetrahy-droxypregna-1.4-diene-3-one; Figure S7: Products of hydrocortisone bioconversion by *M. neoaurum* B-3805∆*kstD/*pMami_*k* on TLC plate (a) Visualization of spots under UV_254_. (b) Visualization of spots on the same plate at UV_365_ after staining with MnCl_2_-reagent. H—hydrocortisone, P—prednisolone, 20-OH-H—20-OH-hydrocortisone (11β,17α,20β,21-tetrahydroxypregn-4-ene-3-one), X1–X3—trace products suggested as intermediates of hydrocortisone degradation; Table S1: PCR primer sequences used in this study; Table S2: 20β-reductase activity of growing actinobacterial cells expressing *kstD2*_NS_ towards hydrocortisone; Table S3: Evaluation of the activity of 1(2)-hydrogenation of prednisolone by recombinant *Mycolicibacterium* cells (aged 36 h) bearing the control plasmids without *kstD2*_NS_ insert at 30 °C.
