# Supplementary material for: Reconstruction of the Steroid 1(2)-Dehydrogenation System from Nocardioides simplex VKM Ac-2033D in Mycolicibacterium Hosts
Source: Microorganisms. 2023 Nov 7;11(11):2720. doi: 10.3390/microorganisms11112720 (PMC10672877; doi:10.3390/microorganisms11112720)
Supplement: Supplementary file 1 [file microorganisms-11-02720-s001.zip › microorganisms-2641367-supplementary.pdf]

microorganisms

MDPI

Supplementary Material

Reconstruction of the Steroid 1(2)-Dehydrogenation System from *Nocardioides simplex* VKM Ac-2033D in *Mycolicibacterium* Hosts

Svetlana R. Fufaeva, Dmitry V. Dovbnya, Tanya V. Ivashina, Andrei A. Shutov and Marina V. Donova *

G. K. Skryabin Institute of Biochemistry and Physiology of Microorganisms, "Pushchino Scientific Center for Biological Research of the Russian Academy of Sciences", 142290 Pushchino, Russia; sfufaeva@list.ru (S.R.F.); anagoge@rambler.ru (D.V.D.); ivashina@ibpm.ru (T.V.I.); w___w@rambler.ru (A.A.S.)

* Correspondence: donova-marina@rambler.ru

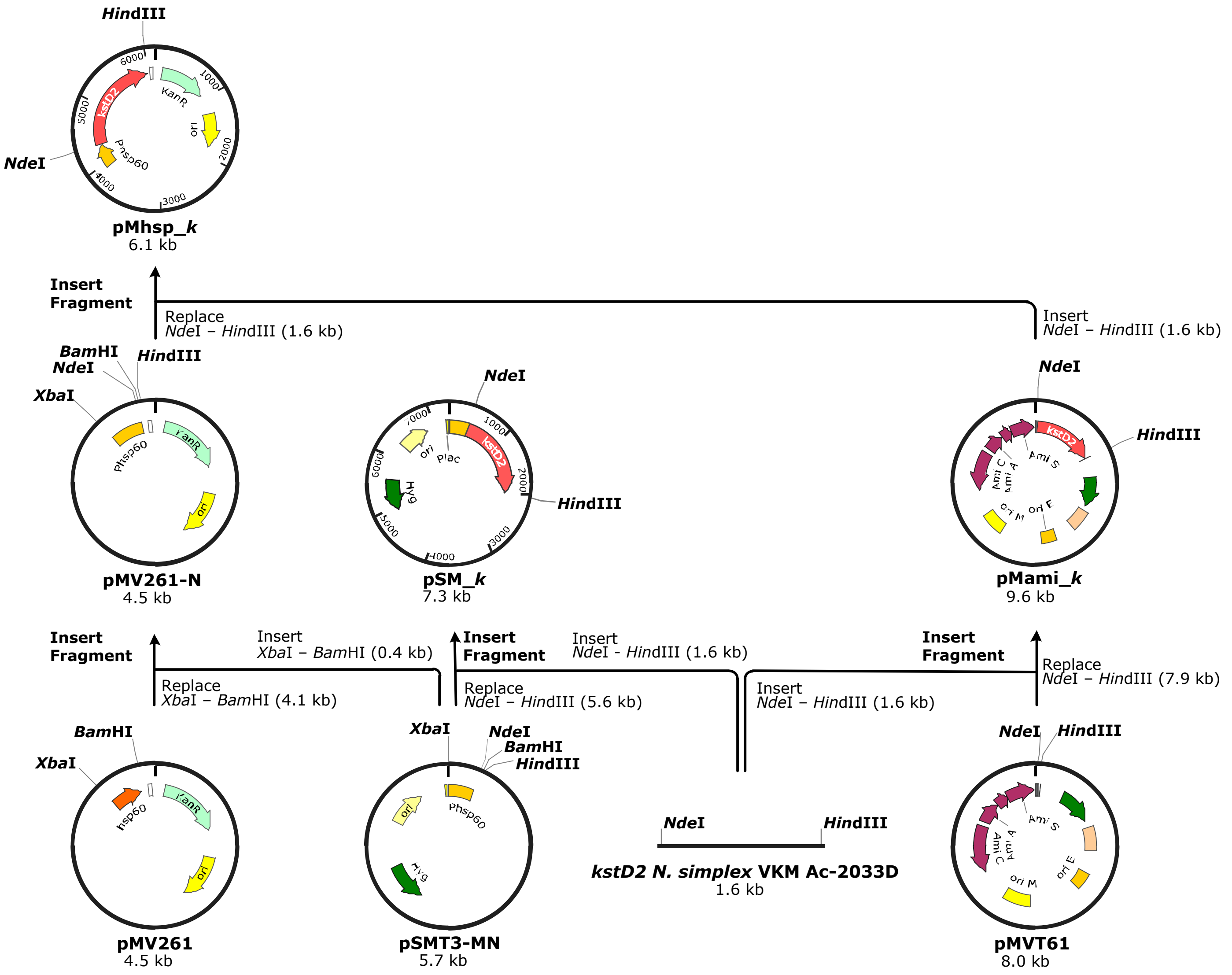

Figure S1. Graphical representation of the construction of recombinant expression plasmids pMhsp_*k*, pMami_*k*, and pSM_*k* containing *kstD2*NS.

Table S1. PCR primer sequences used in this study.

Primer	Sequence 5′→3′	Introduced endonuclease restriction site
kstD2nf	TTATATCATATGTCCGACACCACCGTGGA	*NdeI*
kstD2nr	ATTAAGCTTTCAGGCGGTGGCCGCGT	*HindIII*
kstD2nf2	TTATATCATATGCGAAAGTAACCCGTCATGTCCGACAC	*NdeI*
kstD2nf3	CGTCATGTCCGACACCACCGTGGACCTGC	-
pMVNf	GATGTACGTGGCGAACTCCG	-
pMVNr	CCCAGTCTTTCGACTGAGCC	-
kstD2_1	GACGTCGCTCCAGCTG	-
kstD2_2	TCGACCACGACATGGAC	-
kstD2_3	GTGAACGCGTCCTCGG	-
T1R_r	TCTTTCGACTGAGCCTTTCG	-
Phsp60_f	GCCAGCGTAAGTAGCGG	-

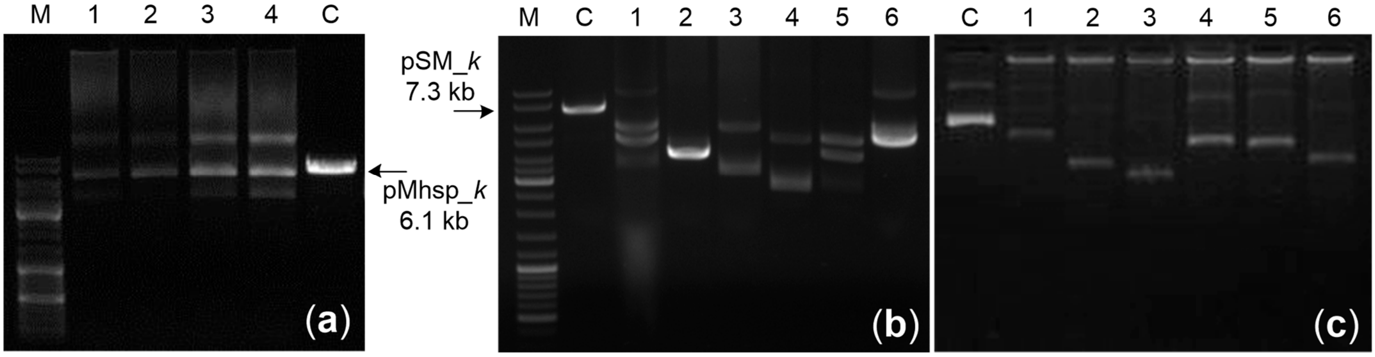

Figure S2. Analysis of plasmid DNA isolated from *Mycolicibacterium* Km^R-transformants. (**a**) *Nde*I-site linearized plasmid DNA: from individual Km^R-clones of *M. smegmatis* BD electroporated with pMhsp_*k* (lanes 1 – 4); original plasmid pMhsp_*k* from *E. coli* used for electroporation (lane C). (**b**) *Hin*dIII-site linearized plasmid DNA: pSM_*k* from *E. coli* used for electroporation (lane C); from individual Hyg^R-clones of *M. smegmatis* BD electroporated with pSM_*k* (lanes 1 – 6). (**c**) Native plasmid DNA: pSM_*k* from *E. coli* used for electroporation (C); from individual Hyg^R-clones of *M. neoaurum* electroporated with pSM_*k* (lanes 1 – 6). M – DNA ladder (Thermo Fisher Scientific, USA).

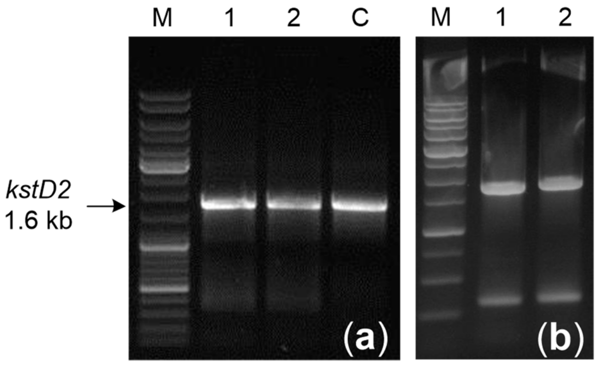

Figure S3. Confirmation of the presence of the *kstD2*$_{NS}$ gene insert (1.6 kb) by PCR analysis. (**a**) Individual Km^R-clones of *M. smegmatis* BD bearing pMhsp_*k* (lane 1) or pMami_*k* (lane 2); C – amplicon from the original plasmid pMhsp_*k* isolated from *E. coli*. (**b**) Individual Km^R-clones of *M. neoaurum* B-3805Δ*kstD*, bearing pMami_*k*. DNA ladder (Thermo Fisher Scientific, USA).

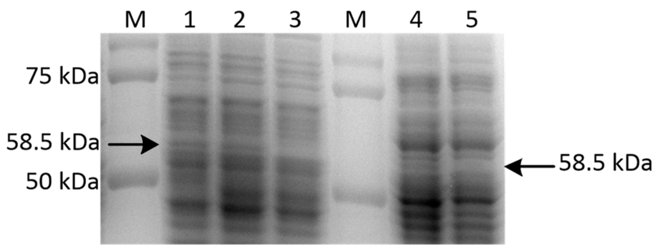

Figure S4. SDS-PAGE analysis of KsdD2 (58.5 kDa) in cell-free extracts of recombinant mycolicibacteria: acetamide-induced *M. neoaurum* B-3805Δ*kstD*/pMami_*k* (lane 1); negative control – acetamide-induced *M. neoaurum* B-3805Δ*kstD*/pMVT61 (lane 2); *M. smegmatis* BD/pMhsp_*k* (lane 3); acetamide-induced *M. smegmatis* BD/pMami_*k* (lane 4); negative control – acetamide-induced *M. smegmatis* BD/pMVT61_*k* (lane 5); M – Protein Ladder (Precision Plus Protein Dual Color Standards, Bio-Rad, USA).

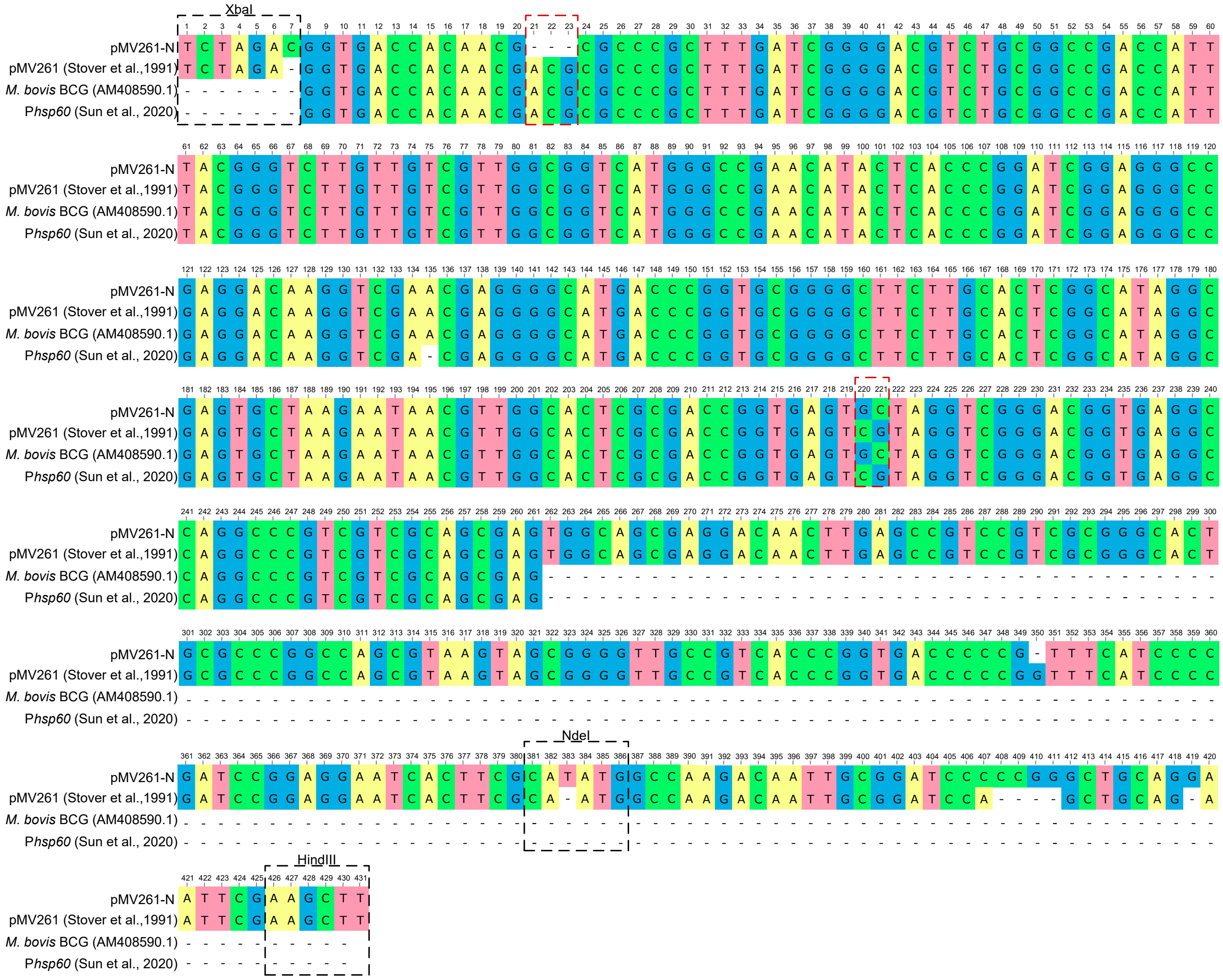

Figure S5. Multiple alignment of nucleotide sequences of the *Xba*I-*Hin*dIII fragment from the plasmids pMV261-N used in this work, pMV261 [41], *hsp60* promoter from *M. bovis* BCG Pasteur 1173P2 (GenBank: AM408590.1), and the sequence of *hsp60* promoter reported by Sun et al., 2020 [49].

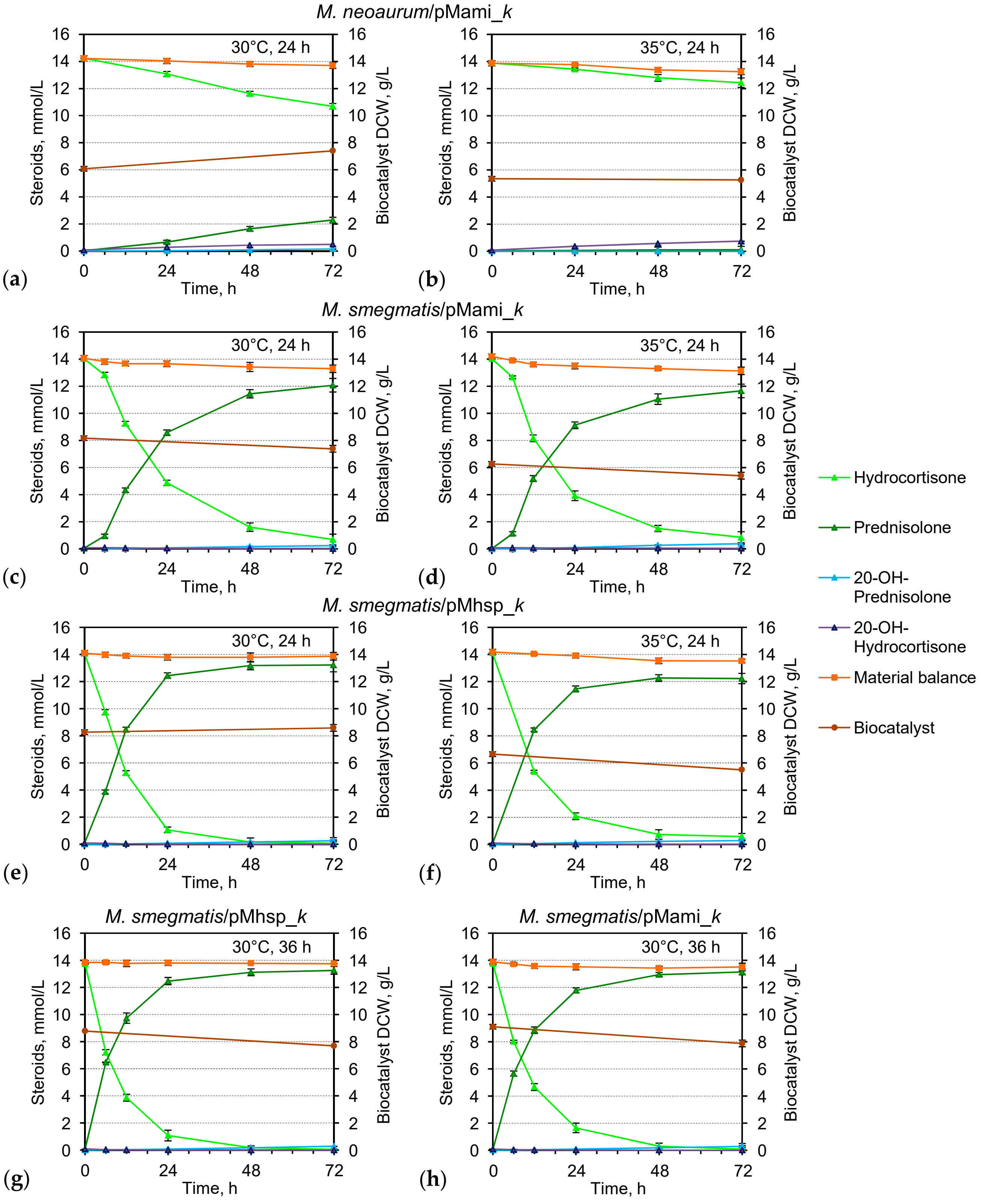

Figure S6. Dynamics of hydrocortisone biotransformation by the cells of *M. smegmatis* BD and *M. neoaurum* B-3805Δ*kstD* bearing experimental plasmids and expressing *kstD2*NS under the control of acetamidase (**a** – **d**, **h**) or *hsp60* (**e** - **g**) promoters at 30°C (**a**, **c**, **e**, **g**, **h**) or 35°C (**b**, **d**, **f**). The cells were cultured in TR3 medium for 24 or 36 h, including 24-h acetamide induction, before the addition of the bioconversion substrate (hydrocortisone, 13.79 mmol/L). 20-OH-Hydrocortisone – 11β,17α,20β,21-tetrahydroxypregn-4-ene-3-one. 20-OH-Prednisolone – 11β,17α,20β,21-tetrahydroxypregna-1,4-diene-3-one.

Table S2. 20β-reductase activity of growing actinobacterial cells expressing *kstD2*$_{NS}$ towards hydrocortisone.

Strains	Bioconversion condition		Max. 20β-reductase activity, μmol/(h×g) (DCW)
	Growth time before substrate addition, h	t, °C	
M. neoaurum B-3805ΔkstD/pMami_k	24	30°C	1.54±0.44
	24	35°C	2.17±0.11
	36	30°C	1.67±0.21
M. neoaurum B-3805ΔkstD/pMVT61	36	30°C	1.83±0.38
	24	35°C	1.94±0.59
M. smegmatis BD/pMami_k	24	30°C	0.41±0.014
	24	35°C	1.52±0.48
	36	30°C	0.48±0.06
M. smegmatis BD/pMVT61	24	35°C	1.12±0.22
	36	30°C	0.31±0.007
M. smegmatis BD/pMhsp_k	24	30°C	0.45±0.019
	24	35°C	0.52±0.02
	36	30°C	0.45±0.009
M. smegmatis BD/pMV261-N	24	35°C	0.95±0.12
	36	30°C	0.22±0.016
N. simplex VKM Ac-2033D	24	30°C	204.1±31.3

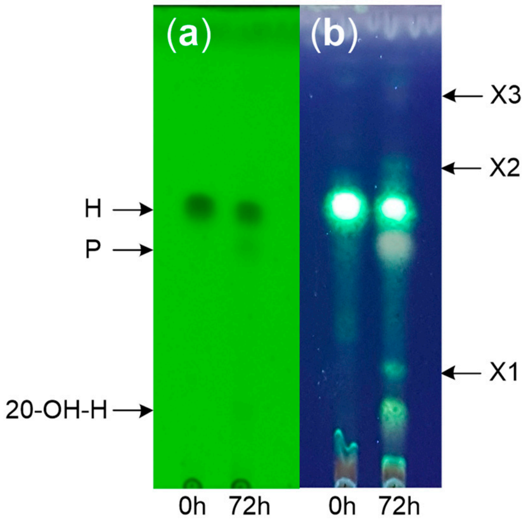

Figure S7. Products of hydrocortisone bioconversion by *M. neoaurum* B-3805Δ*kstD*/pMami_*k* on TLC plate. (**a**) Visualization of spots under UV$_{254}$. (**b**) Visualization of spots on the same plate at UV$_{365}$ after staining with $MnCl_2$-reagent. H – hydrocortisone, P – prednisolone, 20-OH-H – 20-OH-hydrocortisone (11β,17α,20β,21-tetrahydroxypregn-4-ene-3- one), X1 – X3 – trace products suggested as intermediates of hydrocortisone degradation.

Table S3. Evaluation of the activity of 1(2)-hydrogenation of prednisolone by recombinant *Mycolicibacterium* cells (aged 36 h) bearing the control plasmids without *kstD2*$_{NS}$ insert at 30°C.

Mycobacterium strain	Cultivation and bioconversion conditions		Maximal specific steroid 1(2)-hydrogenase activity. μmol/(h×g) (DCW)
	Mixing speed rpm	Induction with acetamide	
M. neoaurum B-3805Δ*kstD*/pMVT61	200	+	0.196±0.015
	100	+	0.208±0.02
M. smegmatis BD/pMVT61	200	+	0.131±0.03
	200	-	0.149±0.025
	100	+	0.134±0.03
